# Systematic screening for unsafe driving due to medical conditions: Still debatable

**DOI:** 10.1186/1471-2458-8-27

**Published:** 2008-01-23

**Authors:** Sandy Leproust, Emmanuel Lagarde, L Rachid Salmi

**Affiliations:** 1 INSERM, U593, Équipe Avenir Santé et Insécurité Routière, 146, rue Léo Saignat, Bordeaux, F-33076, France; 2 IFR99, Université Victor Segalen Bordeaux II, 146, rue Léo Saignat, Bordeaux, F-33076, France; 3 ISPED, Université Victor Segalen Bordeaux II, 146, rue Léo Saignat Bordeaux, F-33076, France; 4 Service d'information médicale, CHU de Bordeaux, Place Amélie Raba Léon, Bordeaux, F-33000, France

## Abstract

**Background:**

Assessing people's ability to drive has become a public health concern in most industrialized countries. Although age itself is not a predictive factor of an increased risk for dangerous driving, the prevalence of medical conditions that may impair driving increases with age. Because the implementation of a screening for unsafe driving due to medical conditions is a public health issue, its usefulness should be judged using standardised criteria already proposed for screening for chronic disease. The aim of this paper is to propose standardised criteria suitable to assess the scientific validity of screening for unsafe driving due to medical conditions, and identify potential issues to be clarified before screening can be implemented and effective.

**Discussion:**

Using criteria developed for screening for chronic diseases and published studies on driving with medical conditions, we specify six criteria to judge the opportunity of screening for unsafe driving due to medical conditions. This adaptation was needed because of the complexity of the natural history of medical conditions and their potential consequences on driving and road safety. We then illustrate that published studies pleading for or against screening for unsafe driving due to medical conditions fail to provide the needed documentation. Individual criteria were mentioned in 3 to 72% of 36 papers pleading for or against screening. Quantitative estimates of relevant indicators were provided in at most 42% of papers, and some data, such as the definition of an appropriate unsafe driving period were never provided.

**Summary:**

The standardised framework described in this paper provides a template for assessing the effectiveness (or lack of effectiveness) of proposed measures for screening for unsafe driving due to medical conditions. Even if most criteria were mentioned in the published literature pleading for or against such a screening, the failure to find quantitative and evidence-based estimates of relevant indicators provides useful insight for further research.

## Background

Road safety has become a major public health concern in European countries, as elsewhere in the developed world [1]. Prevention programmes are most often targeted at the two age groups the most at risk of collision-related injuries, the young and the older drivers [2,3]. Young drivers are at higher risk of collision because of their risky driving behaviours, whereas older drivers seem to be at higher risk of collisions because of functional impairments [4-7]. In a recent study from the United States [8], drivers aged 15 to 24 years had the highest nonfatal injury rate (1934 per 100 million person-trips), followed by those aged 25 to 64 years; drivers older than 65 years had the lowest nonfatal injury rate (600 per 100 million person-trips) but a higher fatal injury rate than middle-aged drivers (15 versus 8 per 100 million person-trips). The apparent over-representation of older drivers in fatal collisions is related to their greater physical frailty and vulnerability to injury when involved in a collision [8]. Although age itself is not a predictive factor of an increased risk for dangerous driving, the prevalence of medical conditions that may impair driving ability increases with age.

To deal with adverse consequences of driving with medical conditions, countries, states or provinces have adopted contrasting policies, including standard periodic renewals of the driving license [9,10], age-based license renewal procedures [6], or, as is the case in France, no policy [1]. These procedures are often simple administrative renewal of the license; in other places applicants must perform tests such as vision tests, or even road tests when specific medical conditions are present [11-13]. Drivers who fail the tests may have their driving privileges revoked, or driving restrictions. However, these official guidelines are often based on expert opinions rather than on documented and critically assessed evidence [14-16].

Because the implementation of screening for unsafe driving due to medical conditions is a public health issue, its usefulness should be judged using guidelines and standardised criteria already proposed for screening for chronic diseases [17]. As in usual screening programmes, indeed, it is important to balance potential positive and negative effects. Moreover, as driving is an essential component of daily living and a major skill potentially needed for independence in everyday adult life, screening measures may have unwanted harmful consequences [1,18,19].

The aim of this paper is to propose standardised criteria suitable to assess the scientific validity of screening for unsafe driving due to medical conditions, and identify potential issues to be clarified before screening can be implemented and effective. Even if the literature is mainly targeted at older drivers, we provide a generalized framework to judge the indication of screening for any medical conditions, age groups, strategies, and types of interventions. We also illustrate the need for such a standard through a survey of published studies pleading for or against screening for unsafe driving, that fail to provide appropriate data.

## Discussion

Using existing criteria developed by Wilson and Jungner in 1968 for screening for chronic diseases [20], then adapted in different fields such as cancer [21], blood transfusion [22], or psychotic disorders [23], and published studies dealing with driving with medical conditions, we specified a general framework providing six criteria to judge the opportunity of screening for unsafe driving due to medical conditions. These criteria are translated into six key questions and related public health indicators.

### Criteria for the indication of screening for unsafe driving

#### Are the consequences of the medical condition on road safety severe enough to move from an individual medical decision making to a systematic screening programme?

**Figure 1:**
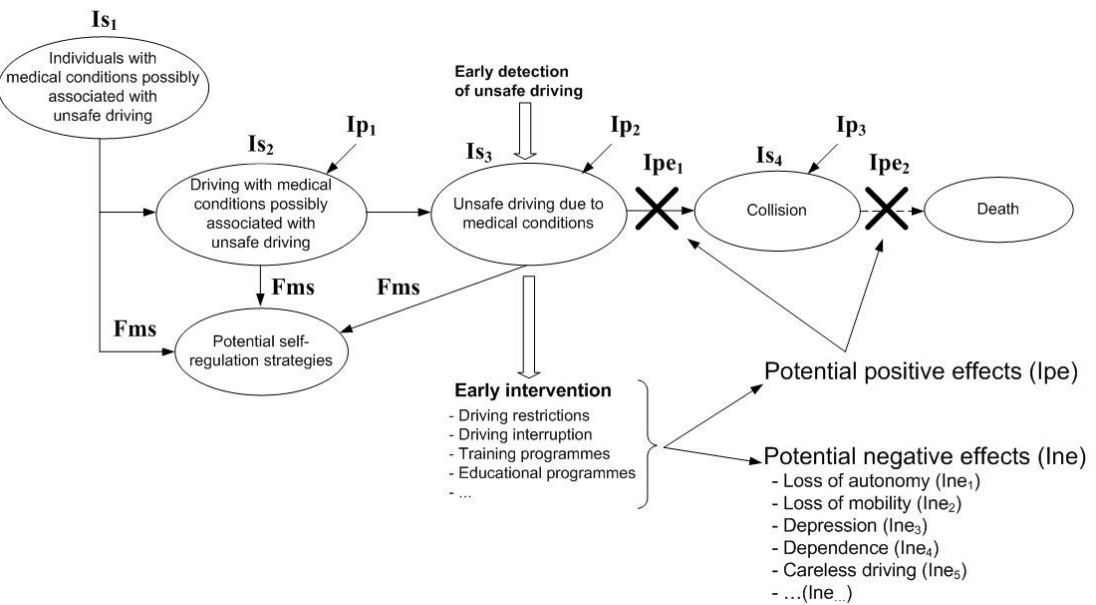
**Natural history and impact of a medical condition on road safety, with and without screening**. Is_1 _to Is_4 _are indicators of severity, Fms is a potential modifier of the indicators of severity; Ipe_1 _and Ipe_2 _are indicators of potential expected positive effects of the early intervention; Ine_1 _to Ine_5 _are indicators of potential expected negative effects of the early intervention; Ip_1 _to Ip_3 _are indicators of performance of screening tools required to detect unsafe driving (see text for details).

To justify a screening programme, there should be: 1/ a high frequency of the medical condition possibly associated with unsafe driving, i.e. a high prevalence in the general population; 2/ a high proportion of individuals with the medical condition who are current drivers; 3/ a high proportion of drivers with the medical condition who really become unsafe drivers; and 4/ a documented higher risk of collision in those drivers who are unsafe because of the medical condition. Estimation of these indicators is difficult as they are strongly related to self-regulation strategies potentially adopted by impaired road users (Figure 1). Some individuals, aware of their diminishing driving abilities, may stop driving, thus do not represent a road safety problem anymore [24]. Other self-regulation strategies involve driving behaviours that allow drivers to remain safe, despite the medical condition [25,26]. For instance, drivers may avoid challenging and at-risk situations (such as left turns) and thus will not be at higher risk of collision [27]. It is thus important to take into account these potential strategies when assessing the actual severity of the consequences of medical conditions on road safety.

#### Is the potentially unsafe driving period long enough to be detected and to implement an effective early intervention?

**Figure 2:**
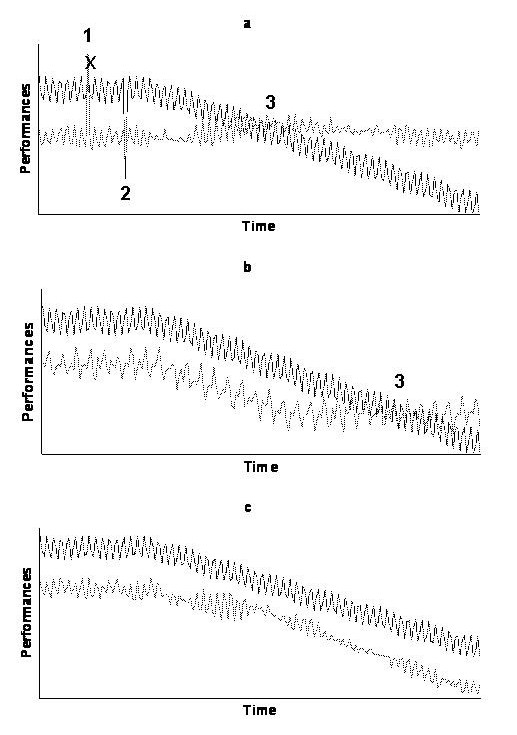
**Hypothetical illustrations of the definition of the potential unsafe driving period**. Dark line is the evolution of the observed performances of the driver; light line is the evolution of the performances needed to drive safely; actual shapes are unknown and hypothetical. a) possible typical situation: the increased risk of collision is due to an acute increase of the performances needed to drive safely (point 1), to an acute decrease of actual performances of the driver (point 2), or because the driving performances of the driver with a medical condition become systematically lower than the performances needed to drive safely (unknown position of point 3). b) the self-regulatory strategies delay the entry in the potential unsafe driving period (shift of point 3). c) the self-regulatory strategies avoid the entry in the potential unsafe driving period.

To implement an early intervention, whatever the context, one must be able to identify a period during which the inability to drive can be detected, and an intervention could be effective. This period, referred to in other contexts as the preclinical phase [17], could be called here the "unsafe driving" period. The unsafe driving period starts when the actual driving performances of the individual become systematically lower, because of the medical condition, than the performances needed to drive safely (Figures 2a and 2b). This period must be long enough to allow early detection of unsafe driving, and implement an early effective intervention, before the lower performances actually result in a collision.

The definition of the unsafe driving period could be based on models of driver behaviour [28] such as the task-capability interface model [29], which takes into account the interaction between task demand and capability. The problem is that many corresponding parameters are unknown. Firstly, we do not know, for most medical conditions, the shape of the evolution of the performances of the driver (capability) [30]. This shape is likely to depend on the natural history of the medical condition, the initial ability of the driver (i.e. driving skills, as influenced for instance by experience), and the driving behaviour (self-regulation strategies to avoid difficult or at-risk situations). Secondly, the shape of the evolution of performances needed to drive safely (task demand) is also unknown as these needs are sensitive to environmental factors, risk exposure, the initial ability of the driver, and his/her driving behaviour. As illustrated in Figure 2a, a driver with a medical condition (or any driver as a matter of fact) could be at higher risk of collision due to an acute increase of the task demand because of environmental factors, or an acute decrease of the capability of the driver, independently of the medical condition. For instance, hearing the bad news can detract from driver competence to yield a somewhat lower level of capability [31]. Another sensible hypothesis is that the driver compensates the risk of collision related to the medical condition by adopting self-regulation strategies and thus decreasing the task demand. This can theoretically postpone (Figure 2b), or completely avoid the entry into the unsafe driving period (Figure 2c). These uncertainties highlight the difficulty to define the starting point of the unsafe driving period, and, consequently, the threshold below which observed performances are actually dangerous, and the appropriate interval between two screens.

#### Is there a reliable and valid test to detect unsafe driving and predict the risk of collision?

A screening programme can be considered only if there is a reliable and valid test during the potential unsafe driving period. The reliability and the validity of the tests are defined as for screening for chronic disease, i.e. respectively by the inter- and intra-observer reproducibility of the test and the sensitivity and specificity in the unsafe driving period. Moreover, the screening test should be easy to administer, brief and cost-effective.

The main difficulty in applying this criterion is the existence of three levels of performances of the test potentially considered (noted Ip_1 _to Ip_3 _in Figure 1). The need for valid and reliable screening tools applies to: 1/the diagnosis of the medical condition; 2/the detection of unsafe behaviours due to this medical condition; and 3/the prediction of the actual risk of collision of a driver diagnosed unsafe. Availability of screening tools for the diagnosis of the medical condition is not the major issue here, as such tests are usually available, for instance for clinical purposes.

The main issue lies in the availability of tools to detect unsafe driving due to the medical condition and to predict the real-condition risk of collision of the driver considered unsafe. In other terms, detection of the medical condition or of so-called unsafe behaviours is only acceptable if these behaviours have been demonstrated to be good proxy markers of the actual risk of collision and its severe consequences [19]. Indeed, the aim of the screening test is not only to distinguish drivers who pass or fail the test but to detect drivers who are really at risk of severe collisions. For instance, when assessing driving ability of older drivers with dementia, there is no test today that is both applicable and clearly and strongly associated with future collisions [19]. For instance, on-road assessment is considered as the "gold standard" evaluation of driving competence for driver with dementia, but it is expensive, based on subjective criteria, and not standardised [19].

Another key issue is whether the best context to assess the ability to drive is the general population of all drivers (systematic screening), or medical contacts for other reasons (case-finding). A recent study of the association between a medical contact and the risk of road-vehicle collisions in older drivers from Quebec indicated that a recent medical contact is associated with an increased risk of crash, thus could represent an opportunity to detect drivers potentially at risk [32].

#### What should be the early intervention for drivers diagnosed unsafe?

The first step is to choose the type of early intervention to impose on drivers diagnosed unsafe. Early interventions most often proposed in the literature range from educational or training programmes to promote safe mobility [33,34] to driving restrictions (geographic areas, hours, type of roads...), including driving cessation [10,35,36]. The latter would be the most logical intervention if the only criterion for judging effectiveness were the impact on the risk of collision. However, the effectiveness of an early intervention is the level to which this intervention meets this objective (number of collisions avoided, possibility to keep driving, and thus to assure mobility), taking into account potential negative effects of this intervention. Indicators of potential positive effects (Ipe_1 _and Ipe_2 _in Figure 1) of an early intervention such as driving cessation could be the number of saved lives or the reduction of the risk of severe injuries since the intervention has been implemented [10,35,36], or, if the intervention is an educational programme, the increase in self-awareness and self-regulatory processes [33,34]. On the other hand, potential negative effects of the intervention (Ine_1 _to Ine_5 _in Figure 1), related to an inappropriate and premature cessation of driving, could be a loss of autonomy or mobility, depression, and the difficulty to access to alternative modes of transportation [19]. Therefore, the main difficulty is to choose the type of intervention to implement according to the published evidence on its potential effectiveness.

#### Would the proposed screening programme result in more good than harm?

Beyond the effect of the intervention in drivers diagnosed unsafe, evaluation of the screening programme should also consider the effect of the programme for all the targeted population, including drivers who are not unsafe. The acceptability of both the considered early intervention (discussed above in the 4^th ^criterion) and of the screening procedures for all drivers targeted should be assessed. Safe and simple screening procedures would be better accepted. For instance, it is unclear whether an assessment of driving abilities using a driving simulator would be better accepted than an on-road driving test. Good validity and reliability of screening tests are also of major importance in the acceptability of the programme, as it is important to minimize the false negatives (falsely reassuring) and false positives (falsely labelling and penalizing). Minimizing the number of false positives is even more important, given that the risk of collision (mostly an issue in truly unsafe drivers) is likely to be lower than the risk of potential adverse consequences (an issue for all positive drivers). These negative consequences (loss of autonomy, need to use other modes of transportation, depression...) would be even less acceptable for drivers wrongly diagnosed unsafe.

#### Would the minimal resources required to implement an effective screening programme be acceptable to the society?

Implementation of an effective and safe screening programme can only be considered if the increase in resources needed to reach the objectives is acceptable to the decision maker and the society. It should consider: 1) the minimal resources requirements needed to maximize the positive effects and minimize the negative effects, including administrative and technical resources (diagnosis of unsafe driving, implementation and follow-up of the early intervention) and human resources (health professionals involved in the process); 2) the costs of implementing all these elements of the screening programme; and 3) whether the expected effects justify the costs using for instance decision analysis methods. Indeed, routine screening with diagnostic tests lacking validity will be costly, as this would target many drivers for further evaluations, such as on-road tests, and would overwhelm existing resources.

### Critical analysis of published articles pleading for or against screening

#### Search and study sampling

Studies potentially eligible were to be published articles in peer-reviewed journals, and to explicitly plead for or against a screening programme to detect unsafe driving due to medical conditions. This information could be provided in the title, the objective, the abstract or the discussion of the article. Potentially eligible studies have been assessed against the inclusion criteria by one reviewer (SL) and, if any doubt, by a second reviewer (LRS).

Electronic searches have been undertaken using MEDLINE, EMBASE, PASCAL and FRANCIS databases to search articles published from January 1985 to December 2006 (Table 1). We considered original studies, editorials or reviews published in English, Spanish or French. We also updated the search using the Safetylit website [37]which provides an updated literature on injury prevention and safety promotion with a special section "transportation issues".

**Table 1:** Search terms used to identify potentially eligible articles

**Database**	**Search terms**
MEDLINE	"accidents, traffic/prevention and control"
	AND "automobile driver examination"
	or
	"accidents, traffic/prevention and control"
	AND "automobile driving" AND "screening"
	or
	"accidents, traffic/prevention and control"
	AND "automobile driving" AND "mass screening"
EMBASE	"automobile driving" AND "screening"
FRANCIS/PASCAL	"vehicle driving" AND "performance evaluation"
	or
	"vehicle driving" AND "medical screening"

The selected studies were then assessed using a standard form listing the criteria for the usefulness of screening for unsafe driving due to medical conditions (Table 2). We counted how many of these studies pleading for or against such a screening programme had mentioned the criteria and how many studies provided relevant quantitative estimates to document these criteria.

**Table 2:** Criteria used in 36 published papers pleading for or against screening for unsafe driving. 36 published papers include 29 original studies, 4 literature reviews and 3 editorials; documented means that quantitative estimates were provided, indicators in parentheses are defined in figure 1

Criteria and corresponding indicators	Mentioned		Documented	
	n	%	n	%
**1. Are the consequences of the medical condition on road safety severe enough?**	**25**	**70**	**14**	**39**
High prevalence of the medical condition *(Is*_1_*)*	15	42	3	11
High proportion of individuals with medical condition who drive *(Is*_2_*)*	10	28	4	11
High proportion of drivers with medical condition who become unsafe *(Is*_3_*)*	0	0	0	0
Higher risk of collision of unsafe drivers due to medical condition *(Is*_4_*)*	21	58	6	17
Frequency of potential self-regulation strategies *(Fms)*	12	33	3	8
**2. Is the potentially unsafe driving period defined and long enough?**	**1**	**3**	**0**	**0**
Definition of a potential unsafe driving period	0	0	0	0
Length of the unsafe driving period	1	3	0	0
**3. Is there a reliable and valid test?**	**24**	**67**	**13**	**36**
Performance of diagnostic test to detect medical conditions *(Ip*_1_*)*	5	14	1	3
Performance of diagnostic test to detect unsafe driving *(Ip*_2_*)*	18	50	12	33
Prediction of collision risk *(Ip*_3_*)*	14	39	6	17
**4. What should be the early intervention for drivers diagnosed unsafe?**	**20**	**55**	**15**	**42**
Nature of intervention	13	36	7	19
Expected positive effects *(Ipe)*	11	30	7	19
Expected negative effects *(Ine)*	12	33	2	5
**5. Would the proposed screening programme result in more good than harm?**	**19**	**53**	**2**	**5**
**6. Would the minimal resources required to implement the screening programme acceptable?**	**8**	**22**	**2**	**5**

#### How criteria are met in published studies pleading for or against screening

Electronic searches and updates yielded 225 potentially eligible studies. Only 62 papers were articles published in peer-reviewed journals, and related to the issue of unsafe driving due to medical conditions. Of these 62 papers, 36 explicitly pleaded for (18 papers) or against screening (18 papers) and were included in the analysis [2,3,5-7,9,10,19,27,30,33-36,38-59]. Almost all studies dealt with screening of cognitive impairments in older drivers. The percentage of papers mentioning relevant criteria ranged from 3% to 70% (Table 2).

#### Consequences of medical conditions on road safety are poorly documented

A majority of papers (70%) mentioned this criterion, and documented both the prevalence of the medical condition and the proportion of individuals with the medical condition who drive, but failed to document the frequency of self-regulation strategies and the impact on road safety. Three studies pleading for screening indicated that the prevalence of dementia ranged from 2 to 8% for people aged 65 years and over, and 80% for 80 years and over [7,19,45]. One study pleading for the usefulness of screening for driver sleepiness indicated that the prevalence of obstructive sleep apnoea (OSA) ranged from 2% to 8% [51].

Two studies pleading for screening documented that almost 30% of patients with dementia are active drivers for approximately 4 years following the diagnosis [19,45]. Only three studies (all pleading for screening) provided information on potential self-regulation strategies [27,38,47] and showed an effect of age on all driving habits: drivers aged 75 and over drive less often at night, and avoid difficult situations [27]. However there was no evidence that these changes in driving habits were related to a lower collision rate [27]. From 22 to 50% of patients with Alzheimer disease stopped driving because of their cognitive problems [38,47]. The proportion of drivers with a medical condition, who were still driving was often mentioned by the authors, but quantitative estimates were lacking. Arguments on self-regulation strategies of older drivers with medical conditions were most often reflecting the opinion of the authors rather than scientific evidence.

No paper distinguished drivers with medical conditions who are still safe drivers from those who really have become unsafe. Quantitative estimates of the risk of collision of drivers with medical conditions were rarely provided: one study pleading for screening of drivers with dementia estimated that the relative risk (RR) of collision was between 2 and 5 compared to non-demented drivers [45]; for young drivers with attention-deficit/hyperactivity disorder (ADHD), one study pleading against screening reported an RR of 1.54 [46]; finally, for male drivers with OSA and aged 36 to 60 years (whose screening was judged indicated by Pierce et al), the reported RR was 3 [51]. For other conditions, no evidence was reported on the relation between these medical conditions and a proven higher risk of collisions.

#### The definition of a potential unsafe driving period is never mentioned nor documented

This was the most important limitation of published studies pleading for or against screening, for any kind of medical condition.

#### The performance of potential screening tools to detect unsafe drivers and predict the risk of collision are poorly documented

The performance of potential screening tools was mentioned in 50% of the papers, but studies failed to document the validity of these tests to detect driving competency and predict the risk of collision. Two studies pleading for screening of older drivers with dementia indicated that the Useful Field Of View (UFOV) was the best developed screening measure for visual attention [19,33]. Dobbs et al also indicated that the Mini Mental State Examination (MMSE) is of questionable utility for predicting driving competence and suggested that the Clinical Dementia Rating (CDR) could be used to assess driving competence [19]. Another study pleading for screening of older drivers with dementia concurred [38]. Finally, Ducheck et al. [39], also pleading for screening, along with Dobbs et al. [19], considered that on-road assessment was the "gold-standard" evaluation to assess driving ability but had several limits. Screening tests proposed by the authors were often screening tools developed to detect the medical condition but with no proven validity to detect driving competency. This is related to the lack of insight in the distinction between the medical condition and the inability to drive.

The prediction of collision risk was considered in almost 40% of the papers but poorly documented. Two studies, one pleading for and one against screening, found that poor performance on neuropsychological tests assessing cognitive abilities is related to future collisions (RR ranging from 1.5 to 3) [56,57], whereas another study, also pleading for screening, claimed that these tests were not sufficiently correlated with future collisions to be valid predictors [19]. One study pleading for screening for visual impairment highlighted that older drivers with a 40% or greater impairment in the UFOV were 2.2 times more likely to be involved in a collision during the 3 years of follow-up [50]. The main weakness of the studies pleading for one test or another was that the predictive value was most often estimated from the correlation with performance on an on-road test or a simulator, without documentation that these results were correlated with driving ability in real condition.

#### The effectiveness of potential early interventions for drivers diagnosed unsafe is poorly documented

Characteristics of the considered early intervention were mentioned in 55% of the studies. Both potential positive and negative effects of the intervention were mentioned in two-thirds of the papers. However, these potential positive effects were poorly documented and quantitative estimates of the incidence of unwanted consequences were scarce.

The best evidence of potential expected positive effects, provided by two studies pleading for screening of older drivers with dementia, was that: 1) an educational intervention could promote self-awareness and self-regulatory processes [33], and 2) restricted licenses to less demanding situations would allow the demented driver with limited competences to drive safely [19]. Another study (against screening) provided quantitative data on the expected positive effect of in-person license renewal, showing that this intervention was associated with a lower fatality rate (RR = 0.83); however vision and road tests were not associated with additional benefits [10]. In the study of Marshall et al. [35], pleading for restricted policies for medical impairments, restricted licensing appeared to decrease rates of traffic violations (RR = 0.93), and collision rates (-12%). In another study pleading for screening, vision policies targeted at drivers 60 years and over were significantly associated with lower fatality rates, with a 12% decrease over a 3-year period [36]. In 2004, Owsley et al. [34] found that an educational programme for visual impairment did not enhance driver safety, moderating the indication of such a screening.

Only two studies provided data on the potential negative effects of the intervention. Dobbs et al. [19], although pleading for screening, argued that using a "co-pilot" is not effective since the driver needs attention both on the road and on the "co-pilot" and thus performs less well; they also argued that driving cessation can be difficult for many patients with dementia and their caregivers who have to deal with the loss of mobility. Finally, in one study pleading against screening, mandatory testing was associated with a higher risk of collision compared to no testing (RR = 1.15) [9].

#### The balance between benefits and risks of the screening programme is almost never documented

The acceptability of the programme was mentioned in 53% of the papers. The study of Hakamies et al. [43] evaluated the potential safety effect of age-related medical screening in Finland, compared to Sweden where no medical screening exists. They demonstrated that age-related variation of vehicle collision and fatality trends was similar in both countries, suggesting that there was no safety-related reason to implement age-based medical screening for older drivers. Moreover, many disadvantages and risks are associated with age-based screening, such as the transfer to riskier travel modes [39] or the induced premature cessation of driving and subsequent immobility [40].

#### The poor feasibility of screening is well documented by two cost-effectiveness analyses

The feasibility and efficiency of the screening programme was only mentioned in 20% of the papers. Two published analyses showed that expected benefits of a screening targeted to older drivers with cognitive impairment are very limited compared to expected costs. In the cost-benefit analysis of Retchin et al. [52], screening for dementia in older drivers every 5 years was associated with a benefit lower than one day of life gain. In the analysis of Viamonte et al. [59], a mass intervention targeted to all older drivers was more cost-effective than a screening programme. None of these analyses considered side effects of screening.

## Summary

The standardised framework described in this paper provides a template for assessing the effectiveness (or lack of effectiveness) of proposed measures for screening for unsafe driving due to medical conditions. Indeed, even if most of the defined criteria were mentioned in the published literature, there is a lack of quantitative and evidence-based estimates of relevant indicators that essentially exist for older drivers with cognitive impairment. Although many official guidelines and recommendations provide, for example in France, a list of medical conditions potentially incompatible with driving (for instance psychotic disorders, excessive sleepiness, severe and permanent cardiac insufficiency...), there is no data, in published studies pleading for or against screening, allowing to judge the need for screening for unsafe driving due to these medical conditions.

Identification of potential issues provides useful insight for further research. Importantly, we need more data on self-regulation strategies potentially adopted by drivers with medical conditions to accurately estimate their real impact on driving exposure and the risk of collision. Although there is evidence that some medical conditions (sleep apnoea, visual impairment, dementia, epilepsy or diabetes) are statistically associated with a higher risk of collision, these associations are usually not clinically meaningful. Most studies considered that having the medical condition (whatever the severity) implied unsafe driving. It is actually difficult to distinguish drivers with the medical condition who become at a clinically higher risk of collision [60-62]. We also dramatically need to document the unsafe driving period. Finally, there is no consensus today on a valid and reliable screening tool to detect unsafe driving and predict the risk of collision in real conditions, and on an early effective intervention, accepted by unsafe drivers and the society. A cohort study would be the best design to get the information needed.

## Competing interests

The author(s) declare that they have no competing interests.

## Authors' contributions

SL and LRS contributed equally to the elaboration of the criteria, evaluation of the literature, drafting and critical revision of the manuscript. EL critically assessed the concepts developed in the manuscript, at several stages of their development. SL, EL and LRS read and approved the final version of the manuscript.

## Pre-publication history

The pre-publication history for this paper can be accessed here:



## References

[B1] White S, O'Neill D (2000). Health and relicensing policies for older drivers in the European union. Gerontology.

[B2] Hakamies-Blomqvist L (2006). Are there safe and unsafe drivers?. Transportation Research Part F: Traffic Psychology and Behaviour Older drivers' safety and mobility: Current and future issues.

[B3] Fitten LJ (2003). Driver screening for older adults. Arch Intern Med.

[B4] Li G, Braver ER, Chen LH (2003). Fragility versus excessive crash involvement as determinants of high death rates per vehicle-mile of travel among older drivers. Accid Anal Prev.

[B5] Meuleners LB, Harding A, Lee AH, Legge M (2006). Fragility and crash over-representation among older drivers in Western Australia. Accid Anal Prev.

[B6] Langford J, Koppel S (2006). The case for and against mandatory age-based assessment of older drivers. Transportation Research Part F: Traffic Psychology and Behaviour Older drivers' safety and mobility: Current and future issues.

[B7] Roche J (2005). Driving and Alzheimer's disease. Psychol Neuropsychiatr Vieil.

[B8] Beck LF, Dellinger AM, O'Neil ME (2007). Motor vehicle crash injury rates by mode of travel, United States: using exposure-based methods to quantify differences. Am J Epidemiol.

[B9] Langford J, Fitzharris M, Koppel S, Newstead S (2004). Effectiveness of mandatory license testing for older drivers in reducing crash risk among urban older Australian drivers. Traffic Inj Prev.

[B10] Grabowski DC, Campbell CM, Morrisey MA (2004). Elderly licensure laws and motor vehicle fatalities. JAMA.

[B11] Physician's Guide to Assessing and Counseling Older Drivers. http://www.ama-assn.org/ama/pub/category/10791.html.

[B12] Assessing Fitness to Drive, 3rd Edition. http://www.austroads.com.au/aftd/index.html.

[B13] Determining Medical Fitness to Operate Motor Vehicles. CMA Driver's Guide, 7th Edition. http://www.cma.ca/index.cfm/ci_id/18223/la_id/1.htm.

[B14] Molnar FJ, Patel A, Marshall SC, Man-Son-Hing M, Wilson KG (2006). Systematic review of the optimal frequency of follow-up in persons with mild dementia who continue to drive. Alzheimer Dis Assoc Disord.

[B15] Molnar FJ, Byszewski AM, Marshall SC, Man-Son-Hing M (2005). In-office evaluation of medical fitness to drive: practical approaches for assessing older people. Can Fam Physician.

[B16] Molnar FJ, Patel A, Marshall SC, Man-Son-Hing M, Wilson KG (2006). Clinical utility of office-based cognitive predictors of fitness to drive in persons with dementia: A systematic review. J Am Geriatr Soc.

[B17] Morrison AS (1992). Screening in Chronic Diseases, 2nd edn.

[B18] Dubinsky RM, Stein AC, Lyons K (2000). Practice parameter: risk of driving and Alzheimer's disease (an evidence-based review): report of the quality standards subcommittee of the American Academy of Neurology. Neurology.

[B19] Dobbs BM, Carr DB, Morris JC (2002). Evaluation and management of the driver with dementia. Neurologist.

[B20] Wilson JMG, Jungner G (1968). The principles and practice of screening for disease.

[B21] Smith R (2003). IARC Handbooks of Cancer Prevention, Volume 7: Breast Cancer Screening. Breast Cancer Res.

[B22] Salmi LR, Mathoulin S, Perez P, Lawson-Ayayi S (1997). [Screening and early detection in blood transfusion: when are they indicated?]. Transfus Clin Biol.

[B23] Cougnard A, Salmi LR, Verdoux H (2003). A decade of debate on early intervention in psychosis: a systematic review of screening criteria. Schizophr Res.

[B24] Freeman EE, Munoz B, Turano KA, West SK (2005). Measures of visual function and time to driving cessation in older adults. Optom Vis Sci.

[B25] Keeffe JE, Jin CF, Weih LM, McCarty CA, Taylor HR (2002). Vision impairment and older drivers: who's driving?. Br J Ophthalmol.

[B26] Cotrell V, Wild K (1999). Longitudinal study of self-imposed driving restrictions and deficit awareness in patients with Alzheimer disease. Alzheimer Dis Assoc Disord.

[B27] Daigneault G, Joly P, Frigon JY (2002). Executive functions in the evaluation of accident risk of older drivers. J Clin Exp Neuropsychol.

[B28] Ranney TA (1994). Models of driving behavior: a review of their evolution. Accid Anal Prev.

[B29] Fuller R (2005). Towards a general theory of driver behaviour. Accid Anal Prev.

[B30] Waller JA (1992). Research and other issues concerning effects of medical conditions on elderly drivers. Hum Factors.

[B31] Lagarde E, Chastang JF, Gueguen A, Coeuret-Pellicer M, Chiron M, Lafont S (2004). Emotional stress and traffic accidents: the impact of separation and divorce. Epidemiology.

[B32] Leproust S, Lagarde E, Suissa S, Salmi LR (2007). Association between road vehicle collisions and recent medical contact in older drivers: a case-crossover study. Inj Prev.

[B33] Bieliauskas LA (2005). Neuropsychological assessment of geriatric driving competence. Brain Inj.

[B34] Owsley C, McGwin G, Phillips JM, McNeal SF, Stalvey BT (2004). Impact of an educational program on the safety of high-risk, visually impaired, older drivers. Am J Prev Med.

[B35] Marshall SC, Spasoff R, Nair R, van Walraven C (2002). Restricted driver licensing for medical impairments: does it work?. Can Med Assoc J.

[B36] Shipp MD (1998). Potential human and economic cost-savings attributable to vision testing policies for driver license renewal, 1989-1991. Optom Vis Sci.

[B37] SafetyLit. http://www.safetylit.org.

[B38] Dubinsky RM, Williamson A, Gray CS, Glatt SL (1992). Driving in Alzheimer's disease. J Am Geriatr Soc.

[B39] Duchek JM, Carr DB, Hunt L, Roe CM, Chengjie X, Shah K, Morris JC (2003). Longitudinal driving performance in early-stage dementia of the Alzheimer type. J Am Geriatr Soc.

[B40] Fitten LJ (1997). The demented driver: The doctor's dilemma. Alzheimer Disease and Associated Disorders.

[B41] Fitten LJ, Perryman KM, Wilkinson CJ, Little RJ, Burns MM, Pachana N, Mervis JR, Malmgren R, Siembieda DW, Ganzell S (1995). Alzheimer and vascular dementias and driving : a prospective road and laboratory study. JAMA.

[B42] Gurubhagavatula I, Maislin G, Nkwuo JE, Pack AI (2004). Occupational screening for obstructive sleep apnea in commercial drivers. Am J Respir Crit Care Med.

[B43] Hakamies-Blomqvist L, Johansson K, Lundberg C (1996). Medical screening of older drivers as a traffic safety measure : A comparative Finnish-Swedish evaluation study. J Am Geriatr Soc.

[B44] Heikkila VM, Kallanranta T (2005). Evaluation of the driving ability in disabled persons: a practitioners' view. Disabil Rehabil.

[B45] Hopkins RW, Kilik L, Day DJ, Rows C, Tseng H (2004). Driving and dementia in Ontario: a quantitative assessment of the problem. Can J Psychiatry.

[B46] Jerome L, Habinski L, Segal A (2006). Attention-deficit/hyperactivity disorder (ADHD) and driving risk: a review of the literature and a methodological critique. Curr Psychiatry Rep.

[B47] Logsdon RG, Teri L, Larson EB (1992). Driving and Alzheimer's disease. J Gen Intern Med.

[B48] Niveau G, Kelley-Puskas M (2001). Psychiatric disorders and fitness to drive. J Med Ethics.

[B49] O'Neill D (1996). Dementia and driving: screening, assessment, and advice. Lancet.

[B50] Owsley C, Ball K, McGwin G, Sloane ME, Roenker DL, White MF, Overley ET (1998). Visual processing impairment and risk of motor vehicle crash among older adults. JAMA.

[B51] Pierce RJ (1999). Driver sleepiness: occupational screening and the physician's role. Aust N Z J Med.

[B52] Retchin SM, Hillner BE (1994). The costs and benefits of a screening program to detect dementia in older drivers. Med Decis Making.

[B53] Reuben DB, Silliman RA, Traines M (1988). The aging driver. Medicine, policy, and ethics. J Am Geriatr Soc.

[B54] Shipp MD, Penchansky R (1995). Vision testing and the elderly driver: is there a problem meriting policy change?. J Am Optom Assoc.

[B55] Simpson CS, Hoffmaster B, Mitchell LB, Klein GJ (2004). Mandatory physician reporting of drivers with cardiac disease: ethical and practical considerations. Can J Cardiol.

[B56] Staplin L, Gish KW, Wagner EK (2003). MaryPODS revisited: updated crash analysis and implications for screening program implementation. J Safety Res.

[B57] Stutts JC, Stewart JR, Martell C (1998). Cognitive test performance and crash risk in an older driver population. Accid Anal Prev.

[B58] Underwood M (1992). The older driver. Clinical assessment and injury prevention. Arch Intern Med.

[B59] Viamonte SM, Ball KK, Kilgore M (2006). A cost-benefit analysis of risk-reduction strategies targeted at older drivers. Traffic Inj Prev.

[B60] Ellen RL, Marshall SC, Palayew M, Molnar FJ, Wilson KG, Man-Son-Hing M (2006). Systematic review of motor vehicle crash risk in persons with sleep apnea. J Clin Sleep Med.

[B61] Charlton JL, Koppel S, O'Hare M, Andrea D, Smith G, Khodr B, Langford J, Odell M, Fildes B (2004). Influence of chronic illness on crash involvement of motor vehicle drivers.

[B62] Hansotia P, Broste SK (1991). The effect of epilepsy or diabetes mellitus on the risk of automobile accidents. N Engl J Med.

